# Advocating for malaria elimination - learning from the successes of other infectious disease elimination programmes

**DOI:** 10.1186/1475-2875-13-221

**Published:** 2014-06-05

**Authors:** Maxine A Whittaker, Angela J Dean, Arna Chancellor

**Affiliations:** 1 School of Population Health, The University of Queensland, Herston, Australia

**Keywords:** Malaria, Malaria elimination, Disease eradication, Health communication, Public-private sector partnerships, Advocacy, Community engagement, Global health planning international development

## Abstract

Malaria elimination is back on the agenda, but it remains challenging for countries to make the transition from effective control to elimination. Many other infectious diseases have been targeted by globally-coordinated elimination advocacy campaigns, and advocacy has been considered an essential component of the success of other disease elimination programmes. What can the malaria community learn from these successes? A review of infectious disease elimination programmes to identify successful elements of advocacy for disease elimination was undertaken. Key elements are: (i) a global elimination plan, supported by international health bodies; (ii) thorough costings and tools to support the business case; (iii) an approach that is positioned within a development framework; (iv) core elimination advocacy messages; (v) provision of advocacy tools for partners (vi) extensive and effective community engagement; and (vii) strong partnerships. These features provide insights into ‘what works’ in global elimination advocacy. Advocacy is a powerful tool to support the long-term political and financial commitment necessary for malaria elimination. The global malaria community needs to work together, to ensure that the early steps towards the end goal of malaria elimination are taken.

## Background

The World Health Organization (WHO) defines malaria elimination as “ reduction to zero of the incidence of infection caused by a specified malaria parasite in a defined geographical area as a result of deliberate efforts” [1]. This requires ongoing and deliberate efforts to prevent reintroduction of transmission. Eradication is “the permanent reduction to zero of the worldwide incidence of infection caused by human malaria parasites as a result of deliberate efforts” [2]. Current approaches to malaria elimination have been influenced by the earlier Global Malaria Eradication Programme (GMEP), which ran from 1955 to 1969. Although the geographical distribution of malaria was reduced during this programme, technical challenges and the resurgence of malaria in many countries led to a shift away from malaria elimination for many decades [3].

Since 2000, there has been an increasing political drive to eliminate malaria [2]. Reducing the burden of malaria is a central component of the Millennium Development Goals (MDGs) [2]. New tools have become widely available, such as artemisinin-based combination therapy, insecticide-treated bed nets, and rapid diagnostic tests [2]. The Roll Back Malaria partnership has listed elimination as a long-term goal, and donors such as the Bill and Melinda Gates Foundation have made strong calls for malaria elimination.

Donor funding for malaria control has increased substantially during this period, and there has been a significant increase in the proportion of the world’s population living in malaria free areas [4,5]. Since 2007, three countries have been certified as malaria-free, seven countries are currently preventing re-introduction to prepare for certification, and ten countries are currently in the elimination phase [2]. Lessons from the GMEP have highlighted the importance of effective coordination between programmes and partners, and implementing elimination programmes flexibly, with capacity to adapt to changing circumstances [3].

Most malaria-endemic countries have not yet reached WHO criteria for pre-elimination, although some of these may have elimination as a long-term goal [6]. Effective and sustained control is an important prerequisite for elimination. However, the transition from sustained control, once achieved, to elimination demands a shift in focus. It requires significant national commitment, and sustained investment and financial support. Advocacy can accelerate the transition to, and support the sustaining of, malaria elimination via several means. Through coordinated campaigns, advocacy can sustain commitment from both donor and endemic countries, strengthen national ownership and partnerships, and position elimination as a driver of development goals.

Elimination advocacy will be informed by the experiences and best practices identified in countries that have successfully achieved elimination. However, this is not the only source of experience. Many other infectious diseases have been targeted by globally-coordinated elimination campaigns. Advocacy has been vital for achieving political prioritization of some neglected tropical diseases (NTD) and subsequent successes [7]. The ongoing success of polio eradication in India has been partly attributed to effective advocacy [8,9]. These programmes exhibit a number of common features, providing insights into ‘what works’ in global elimination advocacy.

### Review methodology

A selective review of infectious disease elimination programmes to identify successful elements of advocacy for disease elimination was undertaken. This process was inclusive, where programmes were included for review if they had documented positive health impact within a particular region, or if a particular element of programme implementation was reported as successful within the literature. Searches were conducted on peer-reviewed databases (e.g., Medline, Web of Science), elimination programme websites, lead organization websites (e.g., World Health Organization, WHO) and internet search engines (e.g., Google). Articles and reports were initially reviewed by one author, and potential themes related to effective advocacy were identified. These themes were then reviewed and grouped in discussion with all authors. Positive and negative experiences, successes, and challenges were included if they were applicable to advocacy for malaria elimination. These were then framed as a ’recommendation’. Lessons that were applicable only to a single disease or not related to advocacy (e.g., technical challenges related to an emerging drug side effect) were not considered in the current review.

### Developing a global elimination plan

#### Background

Global elimination plans are a powerful tool to move the elimination agenda forward, and support advocacy for implementation of elimination strategies at global and national levels.

#### Experience from other diseases

For example, the global push to eliminate lymphatic filariasis (LF) began in response to a 1997 World Health Assembly (WHA) resolution to eliminate LF by 2020 [10]. Subsequent development of the Global Programme for the Elimination of Lymphatic Filariasis (GPELF) created a momentum that resulted in 34 countries establishing elimination programmes [10].

Similarly, recent reductions in the global burden of leprosy began in response to a 1991 WHA Resolution to “eliminate leprosy as a public health problem” by the end of the millennium, defined as reaching a prevalence of less than 1 leprosy case per 10,000 population [11,12]. Leprosy elimination provides an example of how global planning can have unexpected influences on national programmes. By 2001, the global prevalence of leprosy reached less than 1 case per 10,000. However, because of regional clustering of leprosy prevalence, leprosy remained above this threshold in 14 countries. Leprosy support groups have expressed concern that declaring leprosy elimination at a global level led to the perception that leprosy was no longer a concern, and made it difficult for groups to advocate national leprosy programming in endemic countries [13]. A series of follow-up plans attempted to redress this issue, including the WHO ‘Global Strategy for Further Reducing the Leprosy Burden and Sustaining Leprosy Control Activities: 2006-2010’, and a bi-regional strategy to ‘Sustain Leprosy Services in Asia and the Pacific’. Major reductions in prevalence of leprosy occurred during these periods [13]. Global and regional strategies leveraged national commitment from endemic countries to extend their coverage of leprosy services, and provided a foundation for partnerships between WHO and donors that enabled WHO to provide free multidrug treatment for leprosy [11,14]. At country level, global plans provided an impetus for India to commit to elimination targets. This led to funding partnerships, national campaigns [15], and continued reductions in prevalence [13].

There are multiple elements in the process of obtaining a global consensus on the need to target elimination and developing a strategy for action, from WHA declarations and WHO strategies, through to global plans. Most elimination programmes incorporate several of these processes, making it difficult to disentangle their relative importance. However, effective disease elimination programmes typically involve elimination plans, which have a number of common elements:

•*Clearly-defined targets:* existing elimination plans define ‘elimination’ and identify a specific target, and time frame for achieving this target;

•*Defined strategy:* effective plans provide a defined, evidence-based, technical strategy for working towards elimination, with backing from key stakeholders such as those in the areas of technical support and programme management [16]. For example, GPELF provides an elimination strategy focusing on mass drug administration with defined targets and timeframes [17]. Many plans are accompanied by operational guidelines in areas such as integrated health services, surveillance, or treatment [11,18];

•*Widely endorsed*: ideally, elimination plans should be endorsed by international bodies such as the World Health Assembly and key regional and national bodies [19];

•*Avoid a ‘one size fits all’ approach*: effective plans can target local needs and be adjusted to incorporate changes in technology, or emerging challenges. A factor attributed to the success of polio elimination in India is tailoring of the Global Polio Eradication Initiative (GPEI) to the local context, and combining this with innovative social mobilization approaches [20];

•*Incorporates an endgame plan:* endgame management is a key challenge for elimination programmes, especially maintaining political and financial support as the burden of disease diminishes [4,21,22]. The Polio Eradication & Endgame Strategic Plan 2013–2018 [20] ensures that this period is coordinated globally, and investments and elimination can be sustained in the longer term [23];

•*Promotes international and regional collaboration:* implementing elimination plans in border areas between endemic countries [4,24] require collaboration between countries, balancing national strategies with regional, or globally coordinated plans [25].

#### Applying lessons to malaria

The current global approach for malaria control and elimination is provided in the Global Malaria Action Plan (GMAP). This was developed in 2008, supported by the Roll Back Malaria (RBM) Partnership. The current GMAP objectives are to: (i) reduce global malaria deaths to near zero by end 2015; (ii) reduce global malaria cases by 75% by end 2015; and (iii) eliminate malaria by end 2013 in at least eight to ten new countries (since 2008), including the entire WHO European Region. Although elimination is a component of this plan, the overall emphasis remains on effective control. However, as countries sustain control and become ready to consider elimination, few resources are available to facilitate this transition [26]. A global elimination plan provides a platform for the associated advocacy materials and an impetus for countries to develop national elimination plans. It also assists country and regional partners to leverage necessary political and financial support. Planned revisions to the GMAP are currently being undertaken (GMAP 2) [27]. This provides an opportunity for these lessons and new approaches to elimination to be incorporated into the new GMAP.

### Provide tools to support the business case

#### Background

Elimination programmes require long-term financial investment.

#### Experience from other diseases

For NTD elimination, strong cases for return on investment corresponded with improved funding and programme success. For example, a series of cost-benefit analyses have led to the GPELF being described as a ‘best buy’ in global health, and supported continuing programme implementation [28]. The demonstrated cost-effectiveness of the programme is hailed as a major reason for its success [12]. The Global Polio Eradication Initiative developed an economic case for eradication, comparing cost effectiveness of eradication with two alternatives to eradication [29,30]. The Polio Eradication and Endgame Strategic Plan 2013–2018 incorporated detailed costings for all activities [31]. It was estimated that full implementation of the plan would require a budget of US $5.5 billion, covering vaccination, monitoring and surveillance costs, and infrastructure that will benefit other health programmes. To ensure potential donors have confidence in elimination plans, GPEI regularly invites donors to provide ongoing input [32].

#### Applying lessons to malaria

Much of the economic analyses available to inform investment in malaria programmes has focused on malaria control rather than elimination [33,34]. Costs to achieve elimination may be higher than control, and as the burden of disease diminishes, the cost per life saved, a common metric used for prioritization, becomes greater [35]. As prevalence decreases, more sensitive methods of case detection and surveillance are required which can lead to rising costs [1,21]. The importance of stable financing is highlights by a recent systematic review of 75 malaria resurgence events across 61 countries. This review reported that disruptions in financial support was most common reason for malaria resurgence [22]. Thus, access to clearly-defined cost analyses for both elimination and alternative actions such as maintaining control, is vital for policy-makers considering elimination planning. This information is also important for donors, who increasingly need to focus on results and return on investment, and for those advocating for investment in elimination [34,35]. Although this may be politically challenging, quantifying health and economic benefits, direct and indirect, can facilitate commitment to long-term investment [16]. The Malaria Elimination Group has provided initial case studies for elimination costs [36], but full costings and cost-benefit analyses are not yet available for malaria elimination [34,35].

To advocate for malaria elimination, the malaria community need clearly quantified costs and budgets for elimination programmes, and cost-benefit analyses. These analyses will inform approaches to programme financing and partnerships with donors and policy makers. They will also assist in managing stakeholder expectations and identifying gaps in support [25,35]. The changing culture of development financing means there will be greater emphasis on concepts such as value for money, cost effectiveness, performance-based funding and return on investment [6]. As countries move towards elimination, enhanced regional activities will become more important. Given that the Global Fund allocates only a small amount of its funding to regional proposals, it is likely that advocacy efforts will also need to target financing mechanisms and the structure of funding available [4].

### Position elimination within a development framework

#### Background

There have been significant changes in the landscape for funding and implementing global health programmes: fundraising and resource mobilization for malaria programming no longer occur in isolation from global health and development financing [10,34,37]. ‘Competing’ with other health programmes for funding creates inefficiencies and siloing of resources and expertise. In the current resource-constrained environment, this means that advocates for malaria elimination need to work within a developmental framework [31], positioning malaria elimination as a key driver promoting the achievement of other MDGs [7], such as those relating to maternal and child health (MDG4 and 5). In the longer term, elimination advocacy also needs to be positioned for a post-MDG environment, focusing on equity, universal health coverage and sustainability, and also with greater emphasis on economic return on investment.

#### Experience from other diseases

For example, GPELF is promoted as an opportunity to reduce poverty and support sustainable development [37]. The campaign integrates LF elimination with other disease programmes and MDGs, strengthening of health services and universal health coverage, promoting human resource development (including gendered development), availability of technological advances, support for governance and leadership, international networking, and stimulation of international investment [10,25,28,37]. For example, in the Philippines, LF elimination programmes are seen to provide synergies with programmes that work towards poverty reduction, via improving health care systems and access for poor communities [37]. This approach can reduce competition between health and development programming. It can also ensure ongoing national and community support for elimination programmes, even during periods of low disease burden. In the early stages of polio elimination programmes in India and Pakistan, disparity between well-funded polio services and other health services which were poorly funded contributed to community mistrust in elimination programmes, which hampered programme success [38]. Since this period, polio elimination plans have ensured that elimination programming augments existing health services. The Polio Eradication & Endgame Strategic Plan 2013–2018 [32] specifically budgets for health systems strengthening, ensuring that core elements of elimination activities also support the delivery of diverse health services to children.

#### Applying lessons to malaria

There are many opportunities to integrate malaria elimination advocacy within a development framework and create synergies with the development community. Malaria impacts heavily on MDG 4 (reduce child mortality) and 5 (improve maternal health), and addressing malaria has demonstrated benefits on all causes of child mortality [6]. In addition, reducing the malaria burden can lead to improvements in health care systems [37], poverty [39], and economic development [16]. Well-funded malaria interventions can also be used as a vehicle for strengthening maternal and child health care systems and conversely, the recent growth in child and maternal health funding also provides opportunities to implement integrated malaria elimination interventions within this setting [6,40,41]. Such integration can extend service coverage, address gaps in service delivery, and share costs [40]. Malaria elimination also has the potential to align with other areas of development, such as housing improvements [42], water, sanitation and hygiene (WASH) programmes and climate change adaptation. Placing malaria elimination within the framework of the global development agenda can generate valuable synergies for both funding and programming, and creates the opportunity to ensure that malaria elimination contributes to real improvement in diverse health and social outcomes for communities.

### Develop targeted messages

#### Background

Communication is an important component of advocacy, sustaining necessary financial, political and community support. The landscape for malaria elimination is changing: technological advances continue to emerge, financing models need to operate in development-oriented frameworks, and global planning involves cooperation between diverse partners. Within this landscape, it becomes increasingly important for advocates to identify the core messages, and communicate these effectively to target audiences.

#### Experience from other diseases

Successful elimination programmes have developed effective messaging, generating programme support. ‘Make an Invisible Killer Visible’ supported the agenda for maternal and neonatal tetanus (MNT) elimination. ‘Finish the Fight’ reminded donors and supporters of the importance of the endgame in polio elimination. ‘Give a Human Face to Leprosy’ reminds audiences about the ongoing burden of leprosy in affected countries.

#### Applying lessons to malaria

Advocates of malaria elimination need to develop key elimination messages. RBM provides an example of advocacy messaging ‘Sustain Gains, Save Lives: Invest in Malaria’ [6]. Such messages could be adapted for elimination advocacy, for example ‘Sustain Gains Made, Protect Lives, Invest in Malaria Elimination’. Effective messages need to be: clear compelling and concise; consistent and convincing; simple and direct; and frequently reinforced by a variety of sources [43]. Powerful language can create a sense of urgency, but should not resort to sensationalism or overpromise, as these may diminish the impact of the message and the programme [19]. Impact can be enhanced by combining messages with a human face and a visually interesting campaign [9]. These messages may be developed to target major donors, inspire potential partners or generate political support at various levels.

### Provide communication tools

#### Background

Communications tools can empower those advocating at international, national and local levels.

#### Experience from other diseases

Many effective elimination campaigns have provided numerous tools to partners, enabling them to participate in targeted advocacy efforts: for example, the GPELF was supported by the LF Support Center, which provided a Fundraising and Advocacy Toolkit for its partners. This contains a comprehensive range of advocacy materials, such as ‘Top 10 Communication Messages’, a ‘Checklist for Preparing an Effective News Release’, and ‘10 Tips for Writing Letters to Government Officials’.

#### Applying lessons to malaria

It is difficult to estimate the effectiveness of such tools, and few studies detail their specific uptake and impact. However, provision of templates and key messages can ensure that elimination campaigns utilize consistent messaging. In addition, tools can increase the number of partners participating in advocacy activities, via building capacity or confidence to engage in advocacy. RBM does provide a series of advocacy tools [6,44,45]. However, these are not elimination focused. Although some malaria elimination tools are emerging, these are not advocacy focused; for example, The Elimination Scenario Planning toolkit provides tools to support countries in sub-Saharan Africa which are considering progress towards elimination [46].

### Engage with communities

#### Background

Communities affected by malaria must be supported as active participants in elimination, identifying priorities and influencing local programming approaches. It is well recognized that community involvement and ownership can be important drivers of programme success [23,47,48]. High community involvement was identified as an essential factor enabling malaria to be elimination from the island of Aneityum, Vanuatu [23]. Conversely, loss of community support may lead to donors withdrawing funds and potential collapse of a programme [3,21].

#### Experience from other diseases

During the early 2000s, there were a number of setbacks to polio elimination in India and Pakistan. Children in poor Muslim areas with poor sanitation were more likely to be missed in immunization activities due to poor understanding about the need for repeated vaccinations and emerging suspicions about programme safety [38,49]. Widespread media campaigns and intense social mobilization were initiated in response. Health workers and communicators worked with community and village health authorities, conducted repeated family visits in high-risk areas and advocated with community leaders. Evaluations demonstrate that these activities led to improved attendance at vaccination booths, reduced vaccine refusal rates, and a reduction in wild polio cases [38].

Success of the onchocerciasis elimination programmes in Nigeria is linked to community-ownership [50]. Early drug distribution services faced a number of challenges: mobile health workers (a significant cost burden on the programme) were reluctant to travel to high-risk areas, the timing of community visits was often inconvenient for communities, and treatment benefits were not always well communicated. To address these problems, community-based drug distribution was implemented. Following some success, a *community-directed* strategy was then adopted, where communities received training and made decisions about drug distribution methods. This transformed the programme, with an increase in the number of treatments distributed, and the number of staff trained. Services were scaled up, and extended to address other community needs [50].

#### Applying lessons to malaria

The experience from the polio elimination programme highlights how advocates for elimination can benefit from working with specialists in community engagement, social mobilization and communications. Experience from specialists in this area indicates that time required for effective community engagement is often underestimated - it is important that these processes are given adequate time and budgeting in the design phase. Strategies for engaging communities should be flexible, and adaptable to suit diverse cultural, sociopolitical, geographical and health characteristics of communities. The experience from onchocerciasis elimination programmes highlights the importance of making time available to engage with geographically remote communities, or those with a history of conflict. Although community involvement is promoted by WHO and RBM, Kaneko suggests that “community involvement in malaria elimination … is poorly documented” [23]. Community engagement for malaria elimination can be more challenging than for control. As the burden of disease diminishes, communities may shift their focus to other health problems, eg, dengue in Southeast Asia. Not only do advocates need to raise awareness and create demand and sustained support for elimination activities, engagement also provides a key opportunity to identify community needs, and ensure that advocacy and programming activities are integrated with these needs [23,51].

### Create strong partnerships

#### Background

Strong partnerships are a key feature of effective elimination programmes [52], and ineffective partnerships have been cited as a limitation of the GMEP [3].

### Experience from other diseases

Partnerships typical of effective elimination programmes involve *broad engagement,* comprising major international bodies such as WHO or UNICEF, national ministries of health, major donors, research and technical experts, and NGOs [53]. The partnership to support MNT elimination involves UNICEF, the US Committee for UNICEF, the Bill and Melinda Gates Foundation, Becton Dickinson (BD), WHO, PATH (Programme for Appropriate Technology in Health), United Nations Population Fund, as well major donors. The partnership between UNICEF and P&G Pampers began in 2006, with the UK-based campaign “1 pack = 1 vaccine”. This has expanded to 60 countries, with all partners supporting elimination [54]. Private sector partnerships have generated important benefits for elimination programmes. For example, GPELF partners with two pharmaceutical donors (Merck & Co Inc, and GlaxoSmithKline), which provide significant donations of medications ‘for as long as needed’ to LF-endemic countries [10,28,31].

Another success factor is clear leadership and central coordination via a lead agency. UNICEF has a clear mandate to lead the global partnership for MNT elimination [54]. The Global Guinea Worm Eradication Programme provides an additional example of the importance of partnerships. The early years of this programme lacked clear partner coordination [55,56], and were associated with limited funding and failure to meet programme targets [55,56]. In the last decade, strong coordination between the Carter Center and WHO led to joint advocacy to major donors, resulting in major funding pledges [55,56]. Lead agency facilitation is especially important when there is a large number of partner organizations, with diverse organizational priorities, theoretical frameworks and operating approaches.

### Applying lessons to malaria

The shift from malaria control to elimination and efforts in border regions will require a balance between participating countries setting their own priorities, and regional and multi-country strategies and funding mechanisms [4]. Regional leadership will become increasingly important. The RBM Partnership is a broad-based coalition of more than 500 public and private sector partners, which facilitates and coordinates malaria *control* activities [2,5]. The RBM approach allows countries to set their own priorities, whilst supporting sector-wide approaches. The Asia-Pacific Malaria Elimination Network (APMEN) is an example of a country-led regional collaboration that aims to support countries adopting malaria *elimination* as a national or sub-national goal. APMEN works closely with RBM, global agencies, such as WHO, and many regional partners in academic, development, NGO and private sectors [4]. The new strategic plan for RBM may provide an opportunity for them to gain a mandate to broaden its scope to focus on elimination.

## Conclusion

There have been massive gains in malaria control and there is increasing recognition of the importance of malaria elimination as a long-term goal. Once effective control is sustained, many countries are ready to consider elimination. However, it remains difficult to facilitate the transition to elimination without the long-term political and financial support that elimination requires.

Advocacy is an important tool that the global malaria community should use to support the transition to elimination, and sustain gains made in malaria control. Advocacy can leverage political commitment, create new funding opportunities and support partnerships. These lessons highlight a range of challenges and opportunities for the malaria community. The major challenges relate to obtaining a global consensus to endorse a defined strategy focusing on elimination, and developing tools to support the business case. Economic modelling is required to develop robust cost-benefit modelling that focuses on elimination targets. This is a core need for ongoing elimination advocacy. Some of these lessons involve extending existing efforts. For example, the malaria community has a history of developing messages and communications tools; the current opportunity is to extend this work to incorporate elimination targets. Similarly, there are a number of global and regional malaria partnerships that could provide a platform for elimination advocacy - these partnerships need to be provided with the mandate to focus on elimination, with a clear structure of coordination. Most importantly, these lessons also highlight opportunities for the malaria community to embrace new approaches. Advocates for malaria elimination can work within developmental frameworks - building synergies with other health and social programming - to maximize outcomes from investment and prevent competition for increasingly scarce resources. Engaging effectively with communities is vital for building support and optimizing local implementation that is essential for effective long-term programming.

Malaria elimination is a dynamic processes [25]. Elimination advocacy will need to adapt to new technologies and research findings, emerging successes and challenges, changes in the socio-political landscape of eliminating countries, and changes in global health financing. The global malaria community needs to work together, ensuring the early steps towards the end goal of malaria elimination are taken.

## Abbreviations

APMEN: Asia pacific malaria elimination network; GMAP: Global malaria action plan; GMEP: Global malaria eradication programme; GPEI: Global polio eradication initiative; GPELF: Global programme for elimination of lymphatic filariasis; LF: Lymphatic filariasis; MDG: Millennium development goals; MNT: Maternal and neonatal tetanus; NTD: Neglected tropical disease; RBM: Roll back malaria; WHA: World health assembly; WHO: World health organization.

## Competing interests

MW and AC are part of the APMEN Joint-Secretariat. AC is funded through APMEN.

## Authors’ contributions

MW conceived the study and the design of the study; MW, AC and AD undertook the data collection and analysis; AD and MW drafted the manuscript. All authors read and approved the final manuscript.
